# Validity of the Johns Hopkins Adjusted Clinical Groups system on the utilisation of healthcare services in Norway: a retrospective cross-sectional study

**DOI:** 10.1186/s12913-024-11715-4

**Published:** 2024-10-24

**Authors:** Rannei Hosar, Gro Rosvold Berntsen, Aslak Steinsbekk

**Affiliations:** 1 Department of Public Health and Nursing, Norwegian University of Science and Technology (NTNU), Trondheim, Norway; 2 Norwegian Center for E-Health Research, University Hospital of North Norway, Tromsø, Norway; 3 Institute of Community Medicine, UiT The Arctic University of Norway, Tromsø, Norway

**Keywords:** Population health management, Risk stratification, ACG system, Adjusted clinical groups, General practitioner, Hospital

## Abstract

**Background:**

The Adjusted Clinical Groups (ACG) System is a validated electronic risk stratification system. However, there is a lack of studies on the association between different ACG risk scores and the utilisation of different healthcare services using different sources of input data. The aim of this study was therefore to assess the validity of the association between five different ACG risk scores and the utilisation of a range of different healthcare services using input data from either general practitioners (GPs) or hospitals.

**Methods:**

Registry-based study of all adult inhabitants in four Norwegian municipalities that received somatic healthcare in one year (*N* = 168 285). The ACG risk scores resource utilisation band, unscaled ACG concurrent risk, unscaled concurrent risk, frailty flag and chronic condition count were calculated using age, sex and diagnosis codes from GPs and a hospital, respectively*.* Healthcare utilisation covered GP, municipal and hospital services. Areas under the receiver operating curve (AUC) were calculated and compared to the AUC of a model using only age and sex.

**Results:**

Utilisation of all healthcare services increased with increasing scores in the “resource utilisation band” (RUB) and all other investigated ACG risk scores. The risk scores overall distinguished well between levels of utilisation of GP visits (AUC up to 0.84), hospitalisation (AUC up to 0.8) and specialist outpatient visits (AUC up to 0.72), but not out-of-hours GP visits (AUC up to 0.62). The score “unscaled ACG concurrent risk” overall performed best. Risk scores based on data from either GPs or hospitals performed better for the classification of healthcare services in their respective domains. The model based on age and sex performed better for distinguishing between levels of utilisation of municipal services (AUC 0.83–0.90 compared to 0.46–0.79).

**Conclusions:**

Risk scores from the ACG system is valid for classifying GP visits, hospitalisation and specialist outpatient visits. It does not outperform simpler models in the classification of utilisation of municipal services such as nursing homes and home services and outpatient emergency care in primary healthcare. The ACG system can be applied in Norway using administrative data from either GPs or hospitals.

## Background

Risk stratification in healthcare is the process of categorising and stratifying the population according to their health status, expected concurrent and future healthcare needs and risk of experiencing unwanted outcomes [1–3]. As healthcare data have become more accessible, electronic risk stratification tools based on administrative healthcare data have become increasingly popular [1].

One of the most recognised electronic risk stratification tools, both in clinical practice [4] and in research literature [1], is the Johns Hopkins Adjusted Clinical Groups (ACG) System [5]. The ACG system is a population case-mix system that quantifies morbidity and estimates concurrent and future healthcare utilisation through a range of different risk scores. The minimum input data requirements are age, sex and medical diagnoses [5].

Since it was first described by its developers in 1991 [6], the validity of the ACG system has been extensively assessed in numerous settings and healthcare systems. A review from 2021 concluded that it overall performs well for a wide variety of both future and concurrent outcomes [1]. This includes studies across the Americas [7, 8], Asia [9] and Europe, including the Netherlands [10], Spain [11, 12], UK [13, 14] and Sweden [15, 16]. While the system is currently being used in Norway, no studies assessing its validity in Norwegian healthcare has been identified.

The referenced validation studies have varied in terms of input data sources, statistical validation measures and applied ACG risk scores. The ACG system is compatible with both ICD and ICPC diagnosis codes [5], and studies have applied data from primary [10–16] or specialist healthcare services [17, 18], or a combination of the two [7–9, 19]. Commonly reported statistical validation measures have been the area under the curve (reported as AUCs or c-statistics) [7, 8, 10, 15, 19] and explained variance (R^2^ values) [9, 11–14, 16, 17]. Studies have typically reported on the concurrent or prospective relationship between one [8, 11, 15, 19] or a few [7, 9, 10, 13, 14, 16, 17] risk scores from the ACG system and outcome variables such as mortality [13], total healthcare expenditure [9, 12], primary healthcare expenditure [14, 16] or specific healthcare utilisation such as hospitalisation [8, 19], or number of general practitioner (GP) visits [10].

However, none of these studies have investigated the association between different ACG risk scores and the utilisation of different healthcare services using different sources of input data, and more comprehensive analyses have been called for [10]. Furthermore, if an individual receives a high score in one of the ACG risk scores using diagnosis codes registered by e.g., general practitioners as input data, it is currently not clear whether this score is associated with utilisation of other types of healthcare services.

The overall aim of this study was therefore to assess the validity of the association between different ACG risk scores and the utilisation of a range of different healthcare services. This was done by answering to the following research questions using two different data sources (GPs and hospitals) respectively:

1. Within each level of the ACG risk score named resource utilisation band (RUB), what is the number of contacts with different types of healthcare services?
2. Within levels of the ACG risk scores named unscaled ACG concurrent risk, unscaled concurrent risk, frailty flag and chronic condition count, what is the number of contacts with GP or hospital services?
3. To what extent is the five forementioned ACG risk scores able to correctly classify the level of utilisation of different healthcare services compared to a model using age and sex only?

## Methods

### Study design

This was a registry-based study using routinely collected administrative healthcare data on healthcare utilisation and diagnosis codes for all adult residents in four adjacent municipalities in Central-Norway who had visited a somatic healthcare service in 2013. The municipalities had a total population of 168 285 adult individuals in the study period. The STROBE guideline was used to guide the presentation [20].

### Study setting and the ACG system

The publicly funded Norwegian healthcare system provides universal healthcare to all Norwegian residents [21]. Each resident has a personal regular GP [22]. GPs are gatekeepers to specialist healthcare [23], which is mainly delivered in hospitals with local or regional functions. The most specialised functions are centralised to tertiary care in university hospitals or national specialised centres. Emergency primary healthcare is provided by out-of-hours GPs [21]. The municipalities are responsible for providing residents with necessary healthcare services, including home nursing care, home social care and nursing homes [21]. The private healthcare sector is very limited [21].

The study included four municipalities, of which one is a city with a university hospital. The university hospital is also the local general hospital for the population in these municipalities.

The Johns Hopkins ACG System was developed at the School of Hygiene and Public Health at Johns Hopkins University [6, 24]. The system generates different risk scores and indicators that quantify morbidity and estimate concurrent and future healthcare utilisation. The minimum input data requirements are age, sex and all known medical diagnoses for a set period of time, most commonly one year [5]. Additional input data such as medical prescriptions and previous healthcare utilisation can be added to generate additional risk scores.

The adjusted clinical groups (ACGs) are the terminal groups of the ACG system algorithm [5]. To arrive at these terminal groups, diagnoses are first categorised into 32 aggregated diagnosis groups (ADGs) based on the diagnoses’ likelihood of persistence, expected need and cost of procedures, and associated referral to specialist services, hospitalisation, disability and decreased life expectancy. An individual registered with one of the diagnoses in an ADG is assigned to that ADG. Consequently, one individual might be assigned to up to 32 ADGs. The ACG System then clusters the ADGs into collapsed ADGs (CADGs) with similar severity, likelihood of persistence and types of healthcare services needed. Finally, each individual is assigned to one of 93 mutually exclusive terminal ACGs according to their combination of CADGs and demographic characteristics (age and sex). Individuals with similar expected resource requirements can therefore be placed in different ACGs due to different epidemiological patterns of morbidity.

Certain risk scores such as descriptions of resource utilisation are generated by applying weights derived from the ACG by calculating the mean value of all individuals in a reference population. The weights are relative weights standardised to a mean of 1.0. A fixed set of ACG-weights is available through the software, but local sets of weights can be applied to account for local differences.

### Participants

To be included in this study, the individual had to be ≥ 18 years of age at the end of 2013 and registered with ≥ 1 visit to a GP or somatic hospital service from the 1st of January to the 31st of December 2013. Individuals that had only received mental healthcare in the study period were excluded from the study.

### Data sources

Healthcare registry data were collected from three sources. The Norwegian registry for reimbursement claims provided information on all visits to GPs and out-of-hours GPs. Information on all contacts with the hospital were collected directly from the university hospital (equivalent to their reporting to the Norwegian Patient Registry). The municipalities provided data on use of home nursing services.

The first two data sources included information on diagnosis codes associated with each healthcare service contact, registered as ICPC-2 codes for GP visits and ICD-10 codes for contacts with the hospital. Each reimbursement claim made for a GP visit is required to contain minimum one diagnosis code.

### Variables

Risk stratification was performed using the Johns Hopkins ACG® System Population Health Analytics software version 11.0 [25]. This risk stratification was based on the minimum input data requirements of age, sex and diagnosis codes registered by either a GP or the hospital in 2013. Default cost weights based on an American reference population available through the software were applied. Two separate risk stratifications were performed, one with diagnosis codes registered by GPs (hereby referred to as the GP sample) and one with diagnosis codes registered by the hospital (hereby referred to as the hospital sample).

The study assessed the performance of five risk scores from the ACG system, see Table 1 for list. Descriptions are direct copies from the Adjusted Clinical Groups System Version 11.0 Installation and Usage Guide to ensure coherence with official descriptions. The study applied two unscaled risk scores, meaning that the score is set at an average score of 1.0, and that a score above this indicates a higher-than-average resource utilisation. These risk scores were chosen based on their application in previous studies [1, 16, 26, 27], and to provide some variation in types of risk scores.

**Table 1:** Description of the applied ACG risk assessment variables

Risk score	Description
Resource utilisation band	Aggregations of ACGs based upon estimates of concurrent resource that are used to provide a way of separating the population into broad co-morbidity groupings as follows: 0 – No or only invalid diagnoses 1 – Healthy users 2 – Low 3 – Moderate 4 – High 5 – Very high
Unscaled ACG concurrent risk	An estimate of concurrent resource use associated with a given ACG based on a reference database and expressed as a relative value. Each individual is assigned a weight based on their ACG-code
Unscaled concurrent risk	A concurrent total cost risk for this individual for the observation period. Based upon a regression model against a reference population (with a mean of 1.0), the predicted value is expressed as a relative weight
Frailty flag	A flag for any one of the diagnostic clusters that represent discrete conditions consistent with frailty (e.g., malnutrition, dementia, incontinence, difficulty in walking)
Chronic condition count	A count of EDCs (expanded diagnosis clusters) containing trigger diagnoses indicating a chronic condition with significant expected duration and resource requirements

The descriptions are copied from the Adjusted Clinical Groups System Version 11.0 Installation and Usage Guide

Healthcare utilisation variables were continuous variables concerning number of visits to a GP, out-of-hours GP, total number of contacts with hospital, number of inpatient days, outpatient visits at hospital, day treatment contacts, outpatient visits to contract specialists, number of days receiving home nursing services, home social care, number of days of short-term stay at a municipal institution and days as a nursing home resident.

Both ACG risk scores and healthcare utilisation variables are calculated for the same time period (2013). Our analyses do not predict future healthcare utilisation. We therefore consistently use terminology such as classification, characterisation and association to describe the information content of the ACG-scores in our analysis.

### Variable management

For descriptive analyses, the resource utilisation band was applied as a continuous variable. The unscaled ACG concurrent risk and unscaled concurrent risk are presented as either continuous variables or categorised (0–0.49, 0.5–0.99,1–1.99, 2–3.99, ≥ 4) in accordance with two previous studies [26, 27]. The chronic condition count was categorised into < 3 or ≥ 3 chronic conditions [28]. The frailty flag variable was categorised as yes or no. The healthcare utilisation variables were applied as continuous variables.

For logistic regression analyses, the ACG risk scores were applied directly from the ACG analysis output. Healthcare utilisation variables used in the logistic regressions were categorised into binary variables using the median as the cut-off value. To provide a higher level of detail for number of GP visits in the GP sample and number of hospital contacts in the hospital sample, the number of GP visits was dummy coded into five binary variables witch each of the following groups compared to the rest of the sample; < 3, ≥3 to < 6, ≥6 to < 9, ≥9 to < 12, and ≥12 (e.g. “≥3 to < 6” was coded as 1 and all the others as 0). In the hospital sample, the total number of hospital contacts was dummy coded into four binary variables using the following groups; < 3, ≥3 to < 6, ≥6 to < 9, and ≥9. Variables concerning other healthcare services (outpatient hospital visits, inpatient hospital contacts, day treatment at hospital, visit to contract specialist, home nursing service, home social care, short-term stay in municipal institution, nursing home) were transformed into binary variables with a cut-off of < 1 or ≥ 1 visit/contact/day.

### Statistical methods

All analyses were performed using Stata version 17.0 MP.

The number of contacts the individuals within each level of the risk scores had with different healthcare services is presented using descriptive statistics (mean, median and proportions) according to the categorisation described under “Variable management”.

The ability of each risk score to correctly classify the level of contacts the participants had with different healthcare services in the study period was quantified by logistic regression models and the areas under the receiver operating characteristic curve (AUC). Logistic regression is used to analyse the relationship between one or several independent variables and a dependent binary variable [29]. The results from such regressions can be presented as a graphical plot of a curve showing the trend in data called the receiver operating characteristic curve. This curve is the foundation for calculating the “area under the receiving operator characteristic curve”, commonly denoted as AUC, which is an overall metric of discriminatory ability, or in simpler terms of correct classification. AUC values range from 0.5–1, where 0.5 means that the regression model classifies correctly in 50% of cases and 1 means 100% correct classification. An AUC of e.g. 0.73 indicates a 73% likelihood of correct classification. AUCs higher than 0.7 is generally considered acceptable [30].

The classifying ability of each logistic regression is presented as the AUC value and the change in AUC compared to a regression model based on age and sex only. The dependent variable in each regression analysis was the binary categorised healthcare utilisation variable described under “Variable handling”. The independent variable was either the model with age or sex or one of the ACG models.

## Results

**Fig. 1:**
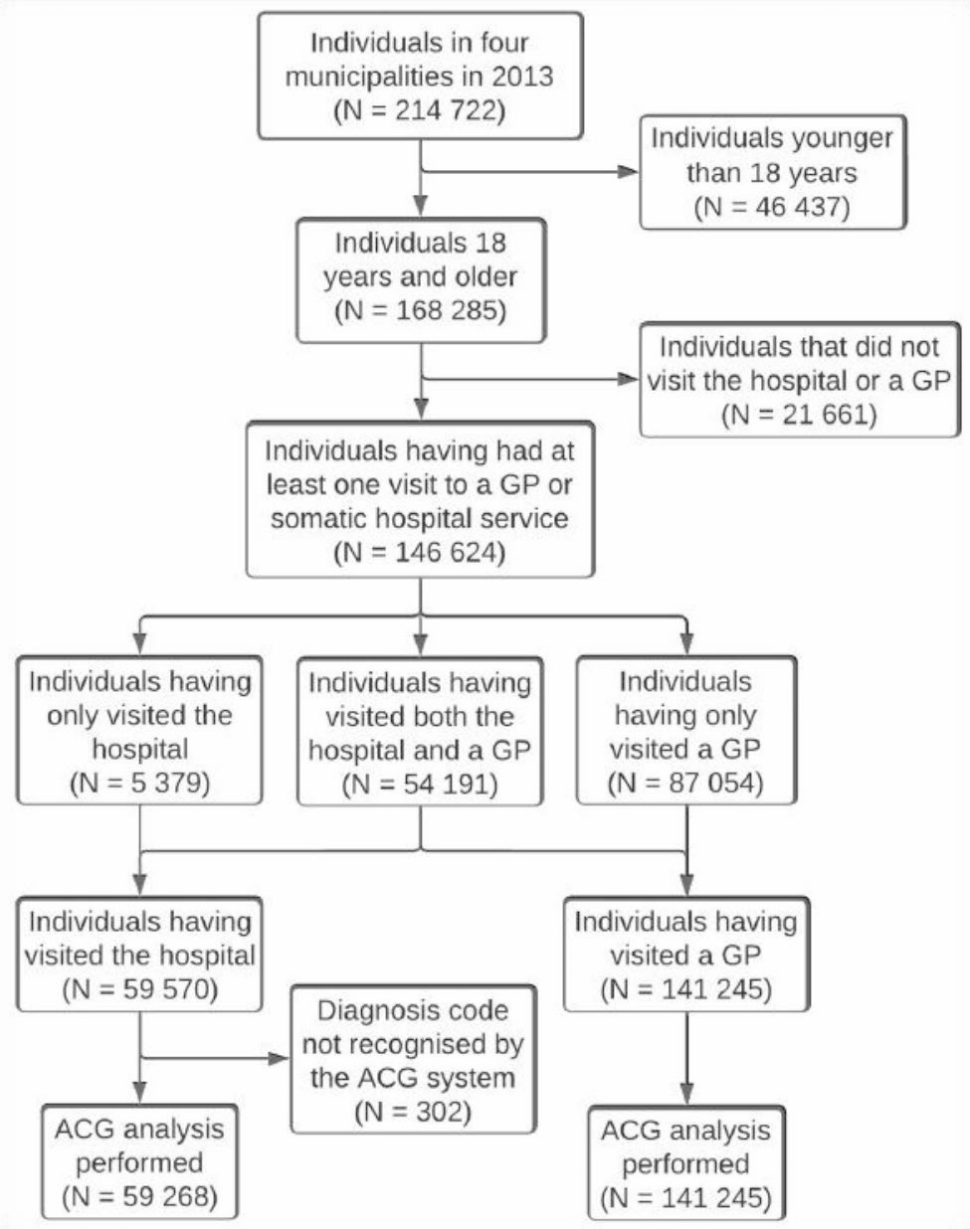
Flow-chart of included participants in four adjacent municipalities in Central-Norway 2013

Of the 168 285 adult residents in the included municipalities in the study period, 146 624 met the criteria for inclusion (Fig. 1). Among these, 141 245 were registered with minimum one valid diagnosis code from a visit to a GP and 59 268 were registered with minimum one valid diagnosis code from a visit to the local hospital. The same individual was present in both samples if registered with a valid diagnosis code from both a GP and the hospital.

The individuals included in the analysis of those having visited a GP (the GP sample) had a mean age of 47 years (SD (standard deviation) 18) and 55.5% were female (Table 2). They had a mean number of 7.3 GP visits and 4.5 hospital contacts in the study period. Those who had visited a hospital (the hospital sample) had a mean age of 51 (SD 20), and 57.9% were female (Table 2). The mean number of GP visits in this group was 9.8 and the mean number of hospital contacts was 4.4. Those who had visited both a GP and the hospital had the same mean age as the hospital sample (51 years), and a slightly higher proportion were female. Their health care utilisation the past year was slightly higher than both the GP and hospital sample except for their mean number of hospital contacts.

**Table 2:** Characteristics of the GP and hospital sample, and of those present in both samples

Characteristic	GP sample *N* = 141 245	Hospital sample *N* = 59 268	Both samples *N* = 54 191
Age group			
18 to 44 years	68 520 (48.5%)	24 569 (41.4%)	21 564 (39.8%)
45 to 64 years	43 829 (31.0%)	17 629 (29.7%)	16 534 (30.5%)
65 to 74 years	16 549 (11.7%)	9 077 (15.3%)	8 675 (16.0%)
75 to 84 years	8 465 (6.0%)	5 344 (9.0%)	5 100 (9.4%)
85 + years	3 882 (2.7%)	2 649 (4.5%)	2 318 (4.3%)
Sex			
Male	63 360 (44.9%)	24 937 (42.1%)	22 079 (40.7%)
Female	77 885 (55.1%)	34 269 (57.9%)	32 112 (59.3%)
Healthcare utilisation past year			
Mean number of GP visits (SD)	7.3 (8.0)	9.8 (9.9)	10.7 (2.2)
Mean number of out-of-hours GP visits (SD)	1.7 (1.9)	1.9 (2.3)	1.9 (2.3)
Mean number of hospital contacts (SD)	4.5 (5.0)	4.4 (7.4)	4.5 (7.2)
≥ 1 inpatient hospital contact	14 272 (10.1%)	15 176 (25.6%)	14 272 (26.3%)
≥ 1 elective hospital contact	48 972 (34.7%)	53 192 (89.7%)	48 851 (90.1%)
≥ 1 emergency care hospital contact	20 639 (14.6%)	22 304 (37.7%)	20 639 (38.0%)
≥ 1 day of municipal home nursing service	5 553 (3.9%)	4 157 (7.0%)	4 000 (7.4%)
≥ 1 day of municipal social care	4 825 (3.4%)	3 468 (5.9%)	3 346 (6.2%)

Numbers are N (%) or mean (standard deviation, SD)

### ACG analysis using diagnosis codes from GPs

**Fig. 2:**
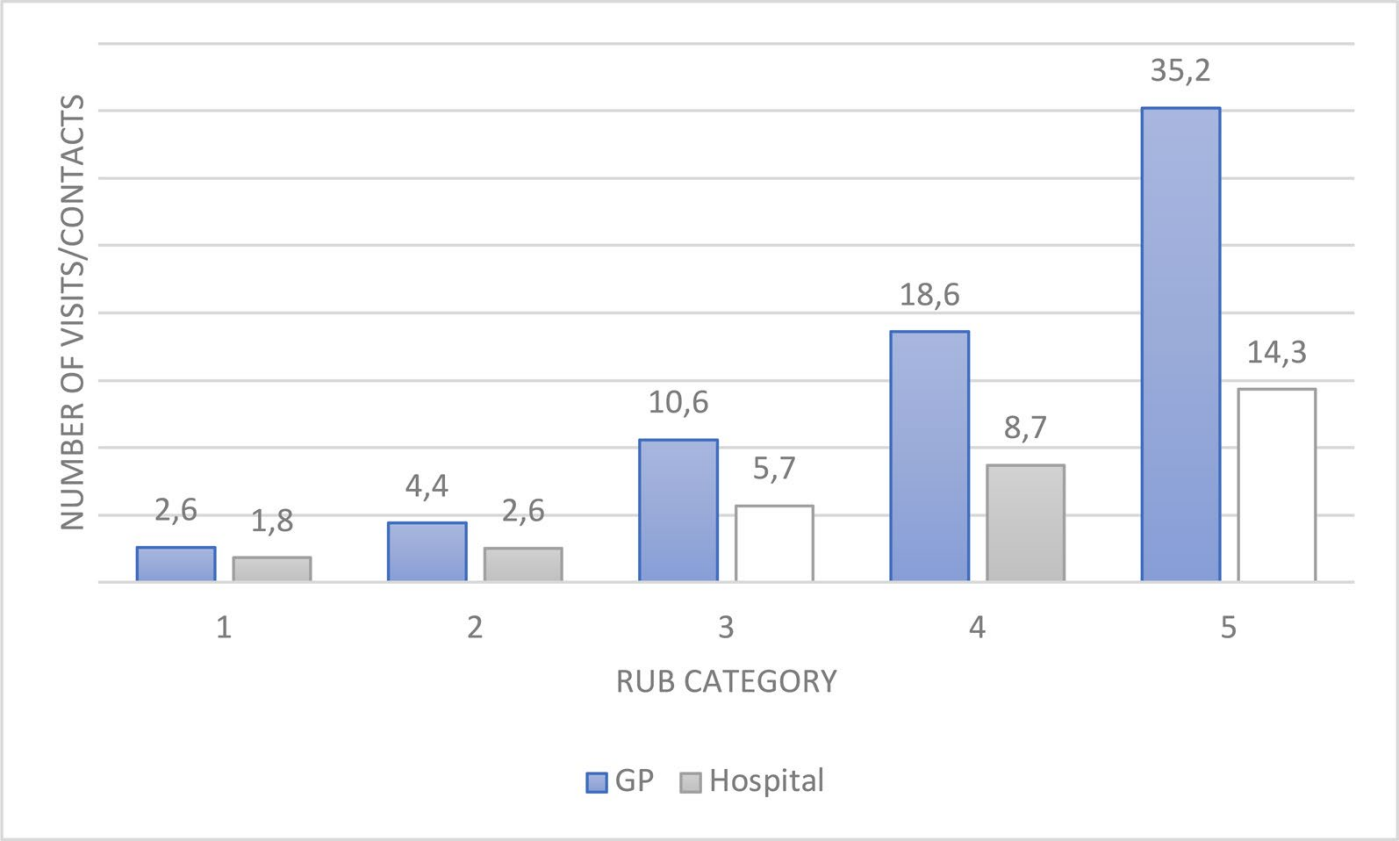
Mean number of visits to a GP (GP sample) or all types of contacts with the hospital (hospital sample) during one year by resource utilisation band (RUB) in four adjacent municipalities in Central-Norway, 2013

The mean and median number of visits to and proportion of the group that had used different healthcare services increased for all types of healthcare services with increasing level of the ACG variable Resource Utilisaton Band (RUB) (Fig. 2, details in Table 3). Those in RUB 1, indicating the lowest estimated resource utilisation, had the lowest number of visits to all healthcare services, whereas those in RUB 5, indicating the highest estimated resource utilisation, had the highest number of visits. The only exception was day treatment in hospital. Comparing those categorised as RUB 1 to those in RUB 5, the number of GP visits increased 11 times (Fig. 2).

**Table 3:** Utilisation of different healthcare services by RUB level in the GP sample

Healthcare service	Resource utilisation band (RUB)				
	1	2	3	4	5
Total	24 495 (17.3)	50 358 (35.7)	62 322 (44.1)	3 907 (2.8)	163 (0.1)
*General practitioner services*					
GP visits					
Mean (SD)	2.6 (2.6)	4.4 (4.3)	10.6 (8.8)	18.6 (13.2)	35.2 (21.3)
Median (range)	2 (1–68)	3 (1–83)	8 (1–150)	15 (1–162)	29 (7–132)
N (% of RUB group)	24 495 (100)	50 358 (100)	62 322 (100)	3 907 (100)	163 (100)
Out-of-hours GP visits					
Mean (SD)	0.2 (0.6)	0.2 (0.7)	0.5 (1.3)	1.0 (2.4)	2.3 (3.5)
Median (range)	0 (0–47)	0 (0–42)	0 (0–63)	0 (0–64)	1 (0–25)
N (% of RUB group)	3 421 (14.0)	8 015 (15.9)	15 847 (25.4)	1 636 (41.9)	104 (63.8)
*Hospital and specialist services*					
Total number of contacts with hospital					
Mean (SD)	0.6 (2.8)	0.9 (3.3)	2.5 (6.0)	6.3 (9.0)	9.6 (9.1)
Median (range)	0 (0–165)	0 (0–188)	1 (0–239)	4 (0–233)	7 (0–47)
N (% of RUB group)	4 859 (19.8)	14 030 (27.9)	31 863 (51.1)	3 396 (86.9)	156 (95.7)
Inpatient days					
Mean (SD)	0.2 (1.8)	0.3 (2.4)	1.2 (5.7)	4.6 (10.0)	13.1 (24.0)
Median (range)	0 (0–80)	0 (0–138)	0 (0–237)	2 (0–180)	3 (0–176)
N (% of RUB group)	939 (3.9)	2 956 (5.9)	10 406 (6.7)	2 237 (57.3)	112 (68.7)
Outpatient visits					
Mean (SD)	0.5 (2.0)	0.7 (4.5)	2.0 (4.8)	4.5 (6.7)	6.9 (7.6)
Median (range)	0 (0–67)	0 (0–67)	0 (0–109)	2 (0–88)	5 (0–40)
N (% of RUB group)	4 179 (17.1)	12 275 (24.4)	28 327 (45.5)	3 084 (78.9)	142 (86.1)
Day treatment contacts					
Mean (SD)	0.0 (1.6)	0.1 (1.6)	0.1 (2.5)	0.3 (4.6)	0.2 (0.4)
Median (range)	0 (0–153)	0 (0–154)	0 (0–199)	0 (0–193)	0 (0–2)
N (% of RUB group)	504 (2.1)	1 471 (2.9)	3 995 (6.4)	392 (10.0)	23 (13.9)
Visits to contract specialist					
Mean (SD)	0.3 (1.6)	0.4 (2.0)	0.8 (2.6)	0.8 (1.9)	1.1 (2.0)
Median (range)	0 (0–69)	0 (0–129)	0 (0–135)	0 (0–39)	0 (0–16)
N (% of RUB group)	3 023 (12.3)	9 069 (18.0)	18 803 (30.2)	1 259 (32.2)	76 (46.1)
*Municipal services*					
Days with home nursing service					
Mean (SD)	1.5 (21.9)	3.5 (33.9)	17.9 (74.9)	44.1 (112.6)	115.2 (159.9)
Median (range)	0 (0–365)	0 (0–365)	0 (0–365)	0 (0–365)	0 (0–365)
N (% of RUB group)	145 (0.6)	644 (1.3)	4 074 (6.5)	625 (16.0)	65 (39.9)
Days with home social care					
Mean (SD)	1.6 (22.9)	3.9 (36.2)	15.9 (71.2)	33.5 (100.0)	62.2 (128.9)
Median (range)	0 (0–365)	0 (0–365)	0 (0–365)	0 (0–365)	0 (0–365)
N (% of RUB group)	153 (0.6)	688 (1.4)	3 482 (5.6)	463 (11.9)	39 (23.6)
Days of short-term stay in municipal institution					
Mean (SD)	0.1 (5.0)	0.3 (8.6)	1.7 (17.9)	3.5 (21.9)	12.6 (42.8)
Median (range)	0 (0–365)	0 (0–365)	0 (0–365)	0 (0–365)	0 (0–322)
N (% of RUB group)	49 (0.2)	208 (0.4)	1 362 (2.2)	224 (5.7)	26 (15.8)
Nursing home residents					
N (% of RUB group)	39 (0.2)	66 (0.1)	121 (0.2)	13 (0.3)	2 (1.2)

Numbers are N (%)

The proportion of each RUB that had utilised each type of healthcare service also increased from RUB 1 to 5 for all services except nursing homes (Table 3). However, the most notable increase is found between RUB 4 and RUB 5, for an example in the increase from a mean number of GP visits of 18.6 in RUB 4 to 35.2 in RUB 5.

There was also a clear trend of increasing mean numbers of GP visits with increasing levels in each ACG risk score (Table 4). The mean number increased nearly eight times from the lowest to the highest unscaled ACG concurrent risk scores and more than three times for the unscaled concurrent risk score. The mean number of GP visits made by individuals flagged as frail were more than double that of those who were not.

**Table 4:** Mean number of GP visits according to ACG risk score in the GP sample

ACG risk score	General practitioner visits
**Unscaled ACG concurrent risk**	
0–0.49	4.2 (4.2, 0.1)
0.50–0.99	9.6 (7.3, 0.0)
1–1.99	16.1 (11.5, 0.1)
2–3.99	18.6 (13.4, 0.2)
≥ 4	32.6 (20.1, 1.4)
**Unscaled concurrent risk**	
0–0.49	5.2 (5.4, 0.0)
0.50–0.99	10.1 (8.4, 0.1)
1–1.99	13.0 (10.5, 0.1)
2–3.99	15.3 (12.5, 0.1)
≥ 4	18.4 (15.7, 0.4)
**Frailty flag**	
No	7.1 (7.7, 0.0)
Yes	15.1 (13.0, 0.2)
**Chronic condition count**	
< 3	6.9 (7.3, 0.2)
≥ 3	20.9 (14.9, 0.0)

Numbers are standard deviation, standard error of the mean. Distribution according to RUB level is provided in Table 3

The ability of five different ACG risk scores to classify levels of contacts with different healthcare services, as measured by AUC, are shown in Table 5. In total, 35 of the 70 AUCs increased using an ACG risk score compared to age and sex alone. The three ACG risk scores concerning concurrent resource utilisation obtained markedly higher AUCs compared to age and sex alone for all healthcare services except the different municipal services (Table 5). The unscaled ACG concurrent risk variable stood out as the ACG variable with the highest AUCs (0.62–0.86) across services. The AUCs of the RUB and unscaled concurrent risk variables were somewhat lower, while the frailty flag and chronic condition count variables showed respectively lower or the same AUC as a model with age and sex only.

**Table 5:** The ability to classify levels of contacts with different healthcare services in the GP sample

Healthcare service	Age and sex	ACG risk score				
		Resource utilisation band (AUC (± change))	Unscaled ACG concurrent risk (AUC (± change))	Unscaled concurrent risk (AUC (± change))	Frailty flag (AUC (± change))	Chronic condition count (AUC (± change))
*General practitioner services (mean value of all AUCs)*	*0.63*	*0.73 (*+ *0.10)*	*0.78 (*+ *0.15)*	*0.71 (*+ *0.08)*	*0.52 (-0.11)*	*0.67 (*+ *0.04)*
GP visits						
≥ 3	0.66	0.80 (+ 0.14)	0.84 (+ 0.18)	0.74 (+ 0.08)	0.51 (-0.15)	0.67 (+ 0.01)
≥ 6	0.67	0.78 (+ 0.11)	0.83 (+ 0.16)	0.74 (+ 0.07)	0.51 (-0.16)	0.69 (+ 0.02)
≥ 9	0.67	0.78 (+ 0.11)	0.84 (+ 0.17)	0.75 (+ 0.08)	0.52 (-0.15)	0.70 (+ 0.03)
≥ 12	0.67	0.77 (+ 0.10)	0.84 (+ 0.17)	0.76 (+ 0.09)	0.52 (-0.15)	0.72 (+ 0.05)
≥ 1 out-of-hours GP visit	0.51	0.60 (+ 0.09)	0.62 (+ 0.11)	0.59 (+ 0.08)	0.51 (= 0.00)	0.55 (+ 0.04)
*Hospital and specialist services (mean value of all AUCs)*	*0.63*	*0.66 (*+ *0.03)*	*0.70 (*+ *0.07)*	*0.67 (*+ *0.04)*	*0.51 (-0.12)*	*0.62 (-0.01)*
≥ 1 hospital contact of any type^a^	0.62	0.67 (+ 0.05)	0.71 (+ 0.09)	0.66 (+ 0.04)	0.51 (-0.11)	0.62 (= 0.00)
≥ 1 outpatient hospital visit	0.62	0.66 (+ 0.04)	0.70 (+ 0.08)	0.65 (+ 0.03)	0.51 (-0.11)	0.61 (-0.01)
≥ 1 inpatient hospital contact	0.64	0.71 (+ 0.07)	0.75 (+ 0.11)	0.70 (+ 0.06)	0.52 (-0.12)	0.65 (+ 0.01)
≥ 1 day treatment at hospital	0.58	0.63 (+ 0.05)	0.66 (+ 0.08)	0.64 (+ 0.06)	0.51 (-0.07)	0.59 (+ 0.01)
≥ 1 visit to contract specialist	0.69	0.61 (-0.08)	0.70 (+ 0.01)	0.69 (= 0.00)	0.51 (-0.18)	0.61 (-0.05)
*Municipal services (mean value of all AUCs)*	*0.87*	*0.68 (-0.19)*	*0.75 (-0.12)*	*0.62 (-0.25)*	*0.59 (-0.28)*	*0.75 (-0.12)*
≥ 1 day of home nursing service	0.84	0.73 (-0.09)	0.79 (-0.05)	0.70 (-0.14)	0.58 (-0.26)	0.79 (-0.05)
≥ 1 day of home social care	0.84	0.70 (-0.14)	0.77 (-0.07)	0.66 (-0.18)	0.57 (-0.27)	0.77 (-0.07)
≥ 1 day of short-term stay in municipal institution	0.89	0.72 (-0.17)	0.80 (-0.09)	0.66 (-0.23)	0.61 (-0.28)	0.80 (-0.09)
Nursing home resident	0.90	0.55 (-0.35)	0.63 (-0.27)	0.46 (-0.44)	0.58 (-0.32)	0.62 (-0.28)

Numbers are the area under a receiver operating characteristic curve (AUC). The number in parentheses is the change in AUC compared to the AUC of the regression model based on only age and sex. A negative number in parentheses indicates that the ACG risk score shows a lower ability to classify correctly compared to the model based on age and sex. A positive number in parentheses indicates a better ability to classify correctly

^a^Both in- and outpatient contacts of all types

Overall, the difference between the AUC of the model based on age and sex, and the ACG risk scores, were lower for municipal services (-0.44 to -0.05) compared to both general practitioner services (-0.16 to + 0.18) and hospital and specialist services (-0.12 to + 0.11) (Table 5).

### ACG analysis using diagnosis codes from hospitals

Using diagnosis codes from the hospital, gave a similar overall picture as the GP sample. The mean and median number of visits to all types of healthcare services were either stable or increased with increasing RUB level (Fig. 2, details in Table 6). The total number of hospital contacts increased 16 times from RUB 1 to 5 (Fig. 2).

**Table 6:** Utilisation of different healthcare services by RUB level in the hospital sample

Healthcare service	Resource utilisation band (RUB)				
	1	2	3	4	5
Number (%)	8 051 (13.6)	22 372 (37.7)	22 244 (37.6)	5 587 (9.4)	1 014 (1.7)
*General practitioner services*					
GP visits					
Mean (SD)	6.3 (7.2)	7.7 (7.7)	11.5 (10.4)	14.1 (12.0)	21.9 (19.1)
Median (range)	4 (0–125)	6 (0–104)	9 (0–150)	11 (1–132)	19 (7–162)
N (% of RUB group)	6 883 (85.5)	20 232 (90.4)	20 866 (93.8)	5 280 (94.5)	956 (94.3)
Out-of-hours GP visits					
Mean (SD)	0.4 (1.0)	0.4 (1.0)	0.6 (1.6)	0.9 (2.2)	2.5 (4.0)
Median (range)	0 (0–28)	0 (0–34)	0 (0–64)	0 (0–46)	1 (0–63)
N (% of RUB group)	2 100 (26.1)	5 908 (26.4)	7 060 (31.7)	2 304 (41.2)	723 (71.3)
*Hospital and specialist services*					
Total number of contacts with hospital					
Mean (SD)	1.8 (2.5)	2.6 (3.4)	5.7 (8.0)	8.7 (11.5)	14.3 (9.4)
Median (range)	1 (1–55)	2 (1–65)	3 (1–208)	6 (1–233)	9 (1–239)
N (% of RUB group)	8 051 (100)	22 372 (100)	22 244 (100)	5 587 (100)	1 014 (100)
Inpatient days					
Mean (SD)	0.1 (0.6)	0.4 (2.1)	1.9 (5.7)	7.6 (11.4)	27.6 (26.5)
Median (range)	0 (0–11)	0 (0–153)	0 (0–203)	4 (0–182)	20 (0–237)
N (% of RUB group)	442 (5.5)	3 263 (14.6)	7 855 (35.3)	5 217 (93.4)	995 (98.1)
Outpatient visits					
Mean (SD)	1.5 (2.4)	2.1 (3.3)	4.6 (6.3)	5.9 (8.2)	8.8 (11.8)
Median (range)	1 (0–55)	1 (0–65)	3 (0–82)	3 (0–91)	5 (0–109)
N (% of RUB group)	6 817 (84.7)	19 328 (86.4)	20 291 (91.2)	4 830 (86.5)	839 (82.7)
Day treatment contacts					
Mean (SD)	0.0 (0.3)	0.1 (0.4)	0.3 (4.5)	0.5 (6.9)	1.8 (13.5)
Median (range)	0 (0–4)	0 (0–12)	0 (0–206)	0 (0–197)	0 (0–195)
N (% of RUB group)	309 (3.8)	2 246 (10.0)	3 446 (15.5)	652 (11.7)	209 (20.6)
Visits to contract specialist					
Mean (SD)	0.7 (2.9)	0.6 (2.2)	0.9 (2.5)	0.6 (1.6)	0.8 (2.2)
Median (range)	0 (0–135)	0 (0–93)	0 (0–93)	0 (0–38)	0 (0–42)
N (% of RUB group)	1 993 (24.8)	5 723 (25.6)	7 440 (33.4)	1 290 (23.1)	330 (32.5)
*Municipal services*					
Days with home nursing service					
Mean (SD)	7.8 (50.5)	7.5 (49.9)	21.7 (81.1)	34.5 (97.0)	131.3 (150.5)
Median (range)	0 (0–365)	0 (0–365)	0 (0–365)	0 (0–365)	47 (0–365)
N (% of RUB group)	231 (2.9)	594 (2.7)	1 920 (8.6)	845 (15.1)	567 (55.9)
Days with home social care					
Mean (SD)	7.1 (49.0)	7.3 (49.3)	19.3 (77.4)	28.9 (90.8)	103.3 (145.9)
Median (range)	0 (0–365)	0 (0–365)	0 (0–365)	0 (0–365)	0 (0–365)
N (% of RUB group)	194 (2.4)	558 (2.5)	1 616 (7.3)	666 (11.9)	434 (42.8)
Days of short-term stay in municipal institution					
Mean (SD)	0.4 (9.9)	0.5 (9.4)	2.4 (21.9)	4.4 (25.9)	22.0 (58.0)
Median (range)	0 (0–365)	0 (0–365)	0 (0–365)	0 (0–365)	0 (0–365)
N (% of RUB group)	47 (0.6)	155 (0.7)	700 (3.1)	399 (7.1)	315 (31.1)
Nursing home residents					
N (% of RUB group)	7 (0.1)	35 (0.2)	63 (0.3)	35 (0.6)	20 (2.0)

Numbers are N (%)

Similar findings were made when investigating the mean number of contacts with the hospital, inpatient days, and number of different wards each patient had been in contact with (Table 7). The total number of contacts with hospital increased nearly six times from the lowest to the highest level in the unscaled ACG concurrent risk score and nearly five times for the unscaled concurrent risk score. The number of contacts with hospital was a little less than doubled for those flagged as frail compared to those who were not.

**Table 7:** Mean number of hospital contacts according to ACG risk score in the hospital sample

ACG risk score	Total contacts	Inpatient days	Outpatient visits	Day treatment contacts
**Unscaled ACG concurrent risk**				
0–0.49	2.6 (3.4, 0.0)	0.4 (2.0, 0.0)	2.1 (3.3, 0.0)	0.1 (0.4, 0.0)
0.50–0.99	5.6 (7.7, 0.1)	1.8 (5.1, 0.0)	4.6 (6.2, 0.1)	0.3 (4.2, 0.0)
1–1.99	6.5 (10.1, 0.1)	2.8 (7.9, 0.1)	5.2 (7.4, 0.1)	0.4 (6.0, 0.1)
≥ 2	8.6 (11.5, 0.2)	7.6 (11.4, 0.2)	5.8 (8.1, 0.1)	0.5 (6.0, 0.1)
≥ 4	14.6 (19.4, 0.6)	27.1 (26.3, 0.8)	9.1 (12.0, 0.4)	1.7 (13.3, 0.4)
**Unscaled concurrent risk**				
0–0.49	2.5 (3.6, 0.0)	0.3 (1.5, 0.0)	2.2 (3.6, 0.0)	0.1 (0.4, 0.0)
0.50–0.99	3.3 (4.1, 0.0)	0.7 (2.5, 0.0)	2.8 (4.0, 0.0)	0.1 (0.5, 0.0)
1–1.99	4.2 (4.6, 0.0)	1.3 (3.9, 0.0)	3.3 (4.4, 0.0)	0.2 (0.6, 0.0)
2–3.99	6.2 (6.2, 0.1)	3.3 (6.1, 0.1)	4.6 (5.8, 0.1)	0.2 (0.6, 0.0)
≥ 4	11.8 (16.6, 0.2)	10.9 (17.8, 0.2)	8.5 (10.5, 0.1)	1.3 (11.9, 0.1)
**Frailty flag**				
No	4.3 (7.1, 0.0)	1.8 (6.6, 0.3)	3.4 (5.6, 0.0)	0.2 (3.5, 0.0)
Yes	7.1 (13.4, 0.0)	10.1 (17.1, 0.4)	4.7 (6.8, 0.2)	0.9 (10.4, 0.2)
**Chronic condition count**				
< 3	3.8 (5.6, 0.0)	1.1 (4.1, 0.0)	3.0 (4.7, 0.0)	0.2 (2.5, 0.0)
≥ 3	11.2 (15.6, 0.2)	11.7 (18.2, 0.3)	8.0 (10.2, 0.1)	1.2 (10.4, 0.1)

Numbers are standard deviation, standard error of the mean. Distribution according to RUB level is provided in Table 3

In total, 28 of the 70 AUCs increased using an ACG risk score compared to age and sex alone (Table 8). The RUB, unscaled ACG concurrent risk and unscaled concurrent risk performed quite similar. The chronic condition count overall performed poorer, and the frailty flag showed no ability to classify the level of contacts with different healthcare services better than a model with age and sex. Overall, the difference between the AUC of the model based on age and sex, and the ACG risk scores, were lower for municipal services (-0.28 to + 0.02) compared to both general practitioner services (-0.14 to + 0.07) and hospital and specialist services (-0.24 to + 0.22).

**Table 8:** The ability to classify levels of contacts with different healthcare services in the hospital sample

Healthcare service	Age and sex	ACG risk score				
		Resource utilisation band (AUC (± change))	Unscaled ACG concurrent risk (AUC (± change))	Unscaled concurrent risk (AUC (± change))	Frailty flag (AUC (± change))	Chronic condition count (AUC (± change))
*General practitioner services (mean value of all AUCs)*	*0.58*	*0.61 (*+ *0.03)*	*0.62 (*+ *0.04)*	*0.59 (*+ *0.01)*	*0.52 (-0.06)*	*0.58 (*= *0.00)*
≥ 8 GP visits	0.65	0.64 (-0.01)	0.66 (+ 0.01)	0.60 (-0.05)	0.51 (-0.14)	0.61 (-0.04)
≥ 1out-of-hours GP visit	0.51	0.57 (+ 0.06)	0.57 (+ 0.06)	0.58 (+ 0.07)	0.52 (+ 0.01)	0.55 (+ 0.04)
*Hospital and specialist services (mean value of all AUCs)*	*0.60*	*0.70 (*+ *0.10)*	*0.73 (*+ *0.13)*	*0.73 (*+ *0.03)*	*0.51 (-0.09)*	*0.66 (*+ *0.06)*
Hospital contact^a^						
≥ 3	0.55	0.75 (+ 0.20)	0.76 (+ 0.21)	0.75 (+ 0.20)	0.51 (-0.04)	0.67 (+ 0.12)
≥ 6	0.55	0.75 (+ 0.20)	0.76 (+ 0.21)	0.77 (+ 0.22)	0.51 (-0.04)	0.70 (+ 0.15)
≥ 9	0.56	0.75 (+ 0.19)	0.77 (+ 0.21)	0.78 (+ 0.22)	0.51 (-0.05)	0.72 (+ 0.16)
≥ 3 outpatient hospital contact	0.75	0.71 (-0.04)	0.72 (-0.03)	0.71 (-0.04)	0.51 (-0.24)	0.68 (-0.07)
≥ 1 inpatient hospital contact	0.59	0.81 (+ 0.22)	0.83 (+ 0.24)	0.81 (+ 0.22)	0.53 (-0.06)	0.67 (+ 0.08)
≥ 1 day treatment at hospital	0.50	0.59 (+ 0.09)	0.57 (+ 0.07)	0.61 (+ 0.09)	0.50 (= 0.0)	0.57 (+ 0.07)
≥ 1 visit to contract specialist	0.69	0.55 (-0.14)	0.68 (-0.01)	0.69 (= 0.00)	0.50 (-0.19)	0.59 (-0.10)
*Municipal services (mean value of all AUCs)*	*0.85*	*0.72 (-0.12)*	*0.77 (-0.08)*	*0.71 (-0.14)*	*0.60 (-0.25)*	*0.76 (-0.09)*
≥ 1 day of home nursing service	0.83	0.72 (-0.11)	0.85 (+ 0.02)	0.85 (+ 0.02)	0.58 (-0.25)	0.77 (-0.06)
≥ 1 day of home social care	0.83	0.70 (-0.13)	0.73 (-0.10)	0.66 (0.17)	0.58 (-0.25)	0.75 (-0.08)
≥ 1 day of short-term stay in municipal institution	0.87	0.77 (-0.10)	0.80 (-0.07)	0.72 (-0.15)	0.63 (-0.24)	0.81 (-0.06)
Nursing home resident	0.88	0.68 (-0.20)	0.71 (-0.17)	0.60 (-0.28)	0.62 (-0.26)	0.70 (-0.18)

Numbers are the area under a receiver operating characteristic curve (AUC). The number in parentheses is the change in AUC compared to the AUC of the regression model based on only age and sex. A negative number in parentheses indicates that the ACG risk score shows a lower ability to classify correctly compared to the model based on age and sex. A positive number in parentheses indicates a better ability to classify correctly

^a^Both in- and outpatient contacts of all types

## Discussion

Overall, this study confirmed findings from previous studies that the ACG system can accurately classify individuals according to their level of healthcare utilisation [6, 8, 10, 12, 14, 17, 19, 24].

We found that the number of contacts with all investigated healthcare services increased with each increasing level of the resource utilisation band (RUB). The number of GP visits and total number of contacts with hospital services also increased with increasing levels of all investigated ACG risk scores. The ability of the different ACG risk scores to distinguish between different levels of utilisation of different healthcare services were overall very good, with some variation between types of service. Among the investigated variables, the unscaled ACG concurrent risk score overall stood out with the highest AUCs and the highest increase in AUC compared to the baseline model. ACG risk scores based on diagnoses from GPs performed better for distinguishing between levels of utilisation of GP visits whereas risk scores based on diagnoses from the hospital performed better for distinguishing between levels of utilisation of different specialist healthcare services. Overall, the ACG risk scores did not outperform a model based on age and sex to distinguish between levels of utilisation of municipal healthcare services.

### Strengths and limitations

The quality and completeness of the applied registries provides a comprehensive overview of relevant diagnosis codes and healthcare utilisation for the population during the study period [31, 32]. Investigating two separate data sources makes the study relevant to different settings and healthcare systems, while also providing new information on the consequences of applying one over the other. Additionally, the use of default American weights in the ACG system without local adaptions increases the generalisability of the study. However, it might be that making local adaptions would improve the accuracy of the system in the Norwegian setting.

However, there are also limitations to consider. Notably, the data applied in the study is from 2013. Although there are no known major changes in coding practices, this represents a limitation. While our aim was to investigate two separate data sources, some integrated healthcare systems would have access to combine data sources and thus benefit from an analysis where these are combined. As the ACG system requires minimum one diagnosis code to generate risk scores, the results describing healthcare utilisation only includes those who visited a GP or hospital in the study period, which yields higher numbers of healthcare utilisation compared to the general population. The study does not report on total cost or cost related to each healthcare visit, and consequently does not account for differences in cost between different e.g. inpatient stays. Further research should look at measures of cost within each type of healthcare service and total cost. Analyses of emergency contacts with hospital that did not result in hospital admission could also be useful. Additionally, previous studies have varied between investigating the association between ACG risk scores and concurrent or future healthcare utilisation. Thus, studies of some types of healthcare services, such as hospitalisations, have only been performed for future healthcare utilisation and cannot directly be compared to the present study. However, investigating the prospective association between ACG risk scores and future healthcare utilisation is still important in its own right.

This study was performed using version 11.0 of the ACG system, while most previous validation studies have applied older versions. As we have not identified published descriptions on which changes have been made to the algorithms in each new version, it is possible that this evolution of the software could cause differing results over time and reduce the comparability between studies that have used different versions of the ACG system.

Other supplemental data such as medical prescriptions could also have been added to generate additional ACG risk scores.

### Associations between the ACG system and nursing home and home service utilisation

Although several studies have assessed the validity of the ACG system for classifying or explaining different outcomes in primary care [1], few studies have assessed the relationship between ACG risk scores and utilisation of municipal services such as nursing homes and home services. Zielinski and Halling concluded that higher RUB categories were associated with receiving more home services in a community-dwelling population in Sweden using an unspecified version of the software and combined input data from both primary and specialist healthcare [33]. Wu et al. found that their indicators of frailty based on the ACG risk scores (version 11, input data from both primary and specialist healthcare and home services), was associated with receiving assistance in instrumental activities of daily living with an AUC of 0.81 [34]. Neither of these studies compared the performance of the ACG system to a model based on age and sex alone, which in the current study was found to outperform the ACG system (Tables 5 and 8). Thus, although the ACG system shows some ability to classify the use of services such as nursing home and home services, there is clearly room for improvement.

One possible explanation for the relatively poor performance of the ACG system compared to the sex and age model might lie in the types of contacts between the elderly and healthcare services, and the coding practices associated with these. A previous description of the frequency of different contacts with GPs for different age groups in Norway [35] shows that individuals over 75 years old and even more so for those over 85 make fewer visits to their GPs, but have higher numbers of simple contacts such as phone calls. Simple contacts have been described to more often be coded as general or administrative diagnosis codes [32], and the codes might thus not accurately reflect the morbidity of each individual. Furthermore, physician services in nursing homes are often provided by dedicated nursing home physicians that do not report diagnosis codes in the same manner as GPs [21].

### Associations between the ACG system and general practice utilisation

Previous studies have assessed the relationship between the ACG system and GP visits, using different versions of the ACG software and different components of the ACG system, input data, statistical analyses and outcome measures [1, 12, 13].

Our analyses using input data from GPs showed AUCs up to 0.84. Similar results have previously been described by Girwar et al. [10], who found AUCs between 0.79 and 0.85 when applying the ADGs and ACGs of the ACG system version 11 based on GP diagnosis codes and pharmaceutical data to distinguish between up to four GP visits. Our results, using the unscaled ACG concurrent risk score directly from the ACG system and without pharmaceutical data as input, was equally precise in classifying more levels of number of GP visits.

Similar findings have been reported by others using other measures than AUC. Sicras-Mainar et al., found the explained variance of the ACGs using ACG version 8.0 based on diagnoses from primary healthcare centres to be 0.53 for number of GP visits [12], and Brilleman et al. found the explained variance of the ACG category to be 0.37 for consultation rates using version 8.2 [13] and input data from GPs. They investigated and compared several of the ACG components for explaining the number of GP visits and found the explained variance to be 0.37 for ACG category, 0.36 for Expanded Diagnosis Clusters (EDCs) and 0.35 for RUB. This finding is interesting, as our study revealed larger differences between the different ACG risk scores in their ability to classify levels of healthcare utilisation. For an example, the ACG unscaled concurrent risk overall showed higher AUCs compared to the RUB category which is made by an aggregation of the ACGs.

Although we have not identified previous studies reporting on the relationship between ACG risk scores and out-of-hours-GP visits, our results yielding the highest AUC of 0.62 shows limited success. A previous study by Haas et al. reported a similar value of AUC 0.67 [19] when applying the ACG system to predict minimum one outpatient emergency department visit. Taken together, this indicates that the ACG system does not perform well in classifying utilisation of outpatient emergency care neither in primary nor secondary care.

### Associations between the ACG system and hospital and specialist utilisation

Comparison to previous studies on hospital services is also challenging due to differences between studies. One study from the US using an unspecified software version and diagnosis codes from primary and specialist healthcare found the ACG system to have AUCs ranging from 0.67–0.73 when predicting inpatient hospitalisations [19], whereas Lemke et al. obtained an AUC of 0.8 for predicting a future inpatient hospitalisation using the EDCs (Expanded Diagnosis Cluster) based on combined input data from both primary and specialist healthcare and pharmacological data in the ACG system version 8.2 [8]. Both studies used input data from one year and utilisation data from the year after. Our study, using input and utilisation data from the same year, got a higher AUC (0.83) using only diagnoses. Furthermore, a recent study from 2023 applied the ACG system version 12.0 with input data on age, sex, registered diagnoses, and prescriptions for a hospital population to evaluate the relationship between different ACG risk scores and inpatient admissions and obtained an AUC of 0.89 when combining the ACG score and a rank probability high total cost score [18]. Olza et al. applied several approaches to predict unplanned inpatient admissions using output from the ACG system version 11.1 based on demographic variables, diagnoses, and pharmaceutical data, and obtained AUCs up to 0.789 to 0.802 [36]. Mentia et al. also obtained high AUCs > 0.8 for an ACG-based model applied to predict unplanned admissions in a general population [37]. In summary, both previous studies and the current study shows that the ACG system performs well for classifying inpatient hospitalisations.

The ACG system was originally developed to classify numbers of outpatient visits [6], and demonstrated to do so in early validation studies [6, 24]. Wahls et al. later found the explained variance of outpatient visits to be 0.30 in a primary care population using version 4.3 of the ACG software [38]. A more recent study found the explained variance of the ACGs for outpatient visits to be 0.113 using the default set cost weights [17]. This corresponds well with our findings based on diagnoses from GPs, while our results based on hospital information were notably higher with AUC values up to 0.83. While there are notable differences in the results from these studies, they overall suggest that the ACG system can correctly classify levels of outpatient visits to hospital services.

### Validity of the ACG system in a Norwegian setting

The current study adds to a number of studies from the past thirty years showing that the ACG system can be applied for different purposes in different contexts.

There is no clear threshold for when a risk stratification model can be considered validated [39]. Previous studies give some indications of when the ACG system has previously been considered applicable in a new setting. For an example, the earliest study stating that the ACG system was statistically valid was written by Weiner et al. in 1991 [6]. They based their conclusion on findings of an explained variance of 0.50 used retrospectively and 0.20 used prospectively in a study population of approx. 160 000 individuals.

More recent studies performed in healthcare systems and populations more directly comparable to the Norwegian setting exist. Halling et al. validated the ACG system in the Swedish primary healthcare setting using polypharmacy as a proxy for total cost in 1 144 individuals [15]. When doing to, they obtained and AUC of 0.72 in their simplest model. A later validation study from Sweden concluded that the ACG system to a high degree explains concurrent primary healthcare costs after obtaining an explained variance of 0.60 and above in a primary care population of approx. 150 000 [16]. One of the most recent studies assessing the performance of the ACG system were carried out by Girwar et al., who concluded that the ACG system is a useful tool in a Dutch general practice context when obtaining AUCs of 0.79–0.85 for distinguishing between numbers of GP visits in a population of 23 618 [10].

Our study included close to 150 000 adults and used two separate data sources, five different ACG risk scores and a range of different outcome measures. Although also performing novel analyses, some of our results are directly comparable to analyses performed in other settings in previous studies as described above. This was achieved without the adjustments used in some other studies, such as local adaptions and local cost weights. Thus, on balance, we argue that this study has shown the validity of the ACG system in a Norwegian context.

### Implications for future practice

In summary, the results of this study can be used by healthcare managers and others involved in healthcare policy formation. One example is that it raises awareness of the choice of data sources. For initiatives using risk stratification to identify individuals utilising specific healthcare services, data from this service ought to be used as the input data source. For initiatives working across different types and levels of healthcare, combining different data sources is recommended. Initiatives aimed specifically at nursing home and home service utilisation appears to have little to no benefit from using the ACG system compares to simpler models. To some extent, this also applies for emergency care.

Regarding choosing which ACG risk score to use, the “unscaled ACG concurrent risk” appears as the overall preferrable choice, but this must be evaluated specifically for each purpose.

## Conclusions

The results from this study show that the number of contacts with different healthcare services consistently increased within each level of the resource utilisation band (RUB), as well as all other investigated ACG risk scores.

The study, compared to other studies on the association between ACG risk scores and health care utilisation, indicates that different risk scores from the ACG system is currently valid for correctly classifying GP visits, hospitalisation and specialist outpatient visits. It does not outperform simpler models in the classification of utilisation of municipal services such as nursing homes and home services and out-of-hours emergency care in primary healthcare. The unscaled ACG concurrent risk score overall obtained the highest AUCs and the highest increase in AUC compared to the baseline model. The ACG risk scores generated using data from either GPs or hospitals performed better for the classification of healthcare services in their respective domains.

Our study demonstrated that the ACG system, used as is with only input of data on age, gender and diagnosis, can be applied as a risk stratification tool in Norway based on administrative data from either GPs or hospitals.

## Data Availability

The dataset supporting the conclusions of this article is available from the corresponding author upon reasonable request.

## Abbreviations

ACG: Adjusted clinical group
ACG system: Johns Hopkins Adjusted Clinical Groups System
AUC: Area under the receiver operating characteristic curve
GP: General practitioner
RUB: Resource utilisation band
EDC: Explanded diagnosis cluster
ADG: Aggregated diagnosis groups
CADG: Collapsed aggregated diagnosis groups
